# Body Surface Area-Based Dosing of Mycophenolate Mofetil in Pediatric Hematopoietic Stem Cell Transplant Recipients: A Prospective Population Pharmacokinetic Study

**DOI:** 10.3390/pharmaceutics15122741

**Published:** 2023-12-07

**Authors:** Hyun Jin Park, Kyung Taek Hong, Nayoung Han, In-Wha Kim, Jung Mi Oh, Hyoung Jin Kang

**Affiliations:** 1 College of Pharmacy and Research Institute of Pharmaceutical Sciences, Seoul National University, Seoul 08826, Republic of Korea; hjpark059@snu.ac.kr (H.J.P.); hanny@jejunu.ac.kr (N.H.); iwkim2@hanmail.net (I.-W.K.); 2 Department of Pediatrics, Seoul National University College of Medicine, Seoul National University Cancer Research Institute, Seoul National University Children’s Hospital, Seoul 03080, Republic of Korea; hongkt@snu.ac.kr; 3 College of Pharmacy, Jeju National University, Jeju 63243, Republic of Korea; 4 Wide River Institute of Immunology, Hongcheon 25159, Republic of Korea

**Keywords:** mycophenolate mofetil, pediatric, population pharmacokinetics, acute graft-versus-host disease, hematopoietic stem cell transplantation

## Abstract

Mycophenolate mofetil (MMF) is commonly used for acute graft-versus-host disease (aGVHD) after allogeneic hematopoietic stem cell transplantation (HSCT). However, limited population pharmacokinetic (PPK) data are available for pediatric HSCT patients. This study aimed to develop a PPK model and recommend optimal oral MMF dosage in pediatric HSCT patients. This prospective study involved pediatric HSCT patients at a tertiary academic institution. Patients received oral MMF 15–20 mg/kg twice daily for aGVHD prophylaxis and treatment. The PPK analysis was conducted using a nonlinear mixed-effects modeling method. Simulation was performed considering different body surface areas (BSAs) (0.5 m^2^, 1.0 m^2^, 1.5 m^2^) and dosing (400 mg/m^2^, 600 mg/m^2^, 900 mg/m^2^ twice daily). Based on the simulation, an optimal dosage of oral MMF was suggested. A total of 20 patients and 80 samples were included in the PPK model development. A one-compartment model with first-order absorption adequately described the pharmacokinetics of mycophenolic acid (MPA). BSA was a statistically significant covariate on V_d_/F. Simulation suggested the optimal dosage of oral MMF as 900 mg/m^2^ twice daily, respectively. A reliable PPK model was developed with good predictive performance. This model-informed optimal MMF dosage in pediatric HSCT patients can provide valuable dosing guidance in real-world clinical practice.

## 1. Introduction

Acute graft-versus-host disease (aGVHD) is a significant complication that results in early and substantial morbidity and non-relapse mortality of patients undergoing allogeneic hematopoietic stem cell transplantation (HSCT) [1,2,3]. Among all patients undergoing HSCT, approximately 30–50% of patients develop aGVHD (grade I–IV), with 14% developing severe aGVHD (grades III–IV) [4]. Notably, patients with severe aGVHD are at high risk of a poor prognosis, with an estimated 1-year overall survival rate of approximately 30–40% [5,6,7]. For the prevention of aGVHD, combinations of tacrolimus, methotrexate, sirolimus, cyclosporine, mycophenolate mofetil (MMF), or post-transplant cyclophosphamide are commonly used as aGVHD prophylactic regimens [8]. Nonetheless, the high incidence rate of aGVHD emphasizes the crucial need for effective management of aGVHD to ensure the success of HSCT recipients.

MMF is an immunosuppressant drug used with calcineurin inhibitors for prophylaxis and treatment of graft-versus-host disease (GVHD) after HSCT as off-label in pediatric and adult patients [9,10,11]. For adult HSCT patients, the recommended MMF dosage is determined to be administered at 10–15 mg/kg twice daily or 1 g twice daily [12,13]. Although dosing regimens for pediatric HSCT patients have been extrapolated from pediatric solid organ transplantation and adult HSCT pharmacokinetic studies [14,15,16,17,18,19,20,21,22,23,24,25,26,27,28,29], there is no standardized dosing regimen for pediatric HSCT recipients due to a lack of clinical evidence. The MMF label suggests dosage based on body surface area (BSA) (600 mg/m^2^) in pediatric solid organ transplant recipients, but in HSCT recipients, dosages vary between BSA-based dosing and weight (kg)-based dosing [9,10,11,30].

Due to the wide inter- and intra-individual pharmacokinetic (PK) variability of MMF and its relatively narrow therapeutic window, therapeutic drug monitoring is required to prevent aGVHD and avoid potential toxicity [31,32]. Therapeutic targets for mycophenolic acid (MPA), which is the active moiety of MMF, have been established with the recommended target area under the curve (AUC)_0–12_ of 30–60 mg·h/L when used with concomitant tacrolimus in solid organ transplantation [14,16,33,34]. This target AUC_0–12_ within the range of 30–60 mg·h/L also has been reported to reduce the incidence of acute and chronic GVHD [11,35,36]. Numerous population pharmacokinetics (PPK) studies have investigated MMF in pediatric solid organ transplantation [14,15,16,18,20,21,28]. In those studies, age, body weight, BSA, time since transplantation, and renal function were identified as notable influencing factors for inter-individual PK difference [14,15,16,18,20,21,28]. However, there is a noticeable lack of PPK-based evidence for pediatric HSCT recipients. HSCT patients have different PK characteristics from solid organ transplant patients [33]; hence, PPK research targeting pediatric HSCT patients is needed. Therefore, the aim of this study was to develop a PPK model for MPA in pediatric HSCT recipients, investigate the PK characteristics of MPA in pediatrics, and suggest optimal MMF dosing strategies for pediatric HSCT recipients.

## 2. Materials and Methods

### 2.1. Study Design and Patient Population

This prospective study was conducted at Seoul National University Hospital from 1 September 2020 to 30 June 2022. Patients aged less than 18 years of age and who had undergone allogeneic HSCT and started MMF for the prophylaxis and treatment of aGVHD were enrolled in the study. Patients with hypersensitivity to MMF or those with concurrent severe infection or unstable vital signs at the time of blood collection were excluded.

The study was approved by the institutional review board of Seoul National University Hospital (IRB No. 2006-120-1133) and was conducted in accordance with the Declaration of Helsinki. Written informed consent was obtained from all participants and their parents or guardians.

### 2.2. Mycophenolate Mofetil Dosing, Blood Sampling, and Data Collection

All patients received oral MMF for the prophylaxis and treatment of aGVHD. The MMF dosage was administered at 15–20 mg/kg twice daily in either oral suspension or capsule formulation [10,13,37]. Whole blood samples were drawn at pre-dose at 0 h and at post-dose 1, 2, 6 h after MMF administration at least 3 days after the initiation of MMF to ensure that the MPA concentration had reached a steady-state concentration. 

Patient demographic characteristics, transplantation information, and clinical information were obtained from the electronic medical records on the day of MMF initiation and on the day of blood sampling. The collected covariates were demographics (age, sex, weight, height, BSA), transplantation information (underlying hematologic disease, conditioning regimen, donor information, ABO matching, post-transplant days, MMF administration days, capsule or suspension formulation of MMF), laboratory values (white blood cell count (WBC), hemoglobin (Hgb), hematocrit (Hct), absolute neutrophil count (ANC), blood urea nitrogen (BUN), serum creatinine (SCr), estimated glomerular filtration rate (eGFR) calculated by Schwartz’s formula, albumin (alb), total bilirubin (t.bil), aspartate transaminase (AST), alanine transferase (ALT), C-reactive protein (CRP), tacrolimus trough level), and other comedication information (fluoroquinolone, azole antifungal agent, proton pump inhibitor, and histamine H2 receptor antagonist). The incidence of aGVHD was prospectively monitored for a period of up to 100 days following HSCT.

Samples were centrifuged at 3000 rpm for 10 min at 4 °C for serum separation. Serum total MPA concentration was determined by using a homogeneous particle-enhanced turbidimetric inhibition immunoassay (PETINIA) technique on a Dimension^®^ EXL 200 (Siemens Healthineers, Erlangen, Germany) [38,39]. The lower limits of quantification were set as 0.1 mg/L.

### 2.3. Pharmacokinetic Analysis

#### 2.3.1. Non-Compartmental Analysis (NCA)

The PK parameters of MPA were estimated using an NCA. NCA was performed using an Excel add-in program, PK solver, R software (Version 4.2.1), and the R packages “NonCompart” and “ncar”. The following PK parameters were determined by an NCA: maximum plasma concentration (C_max_), time to maximum plasma concentration (T_max_), AUC_0–6_, AUC_0–inf_, apparent clearance (CL/F), and apparent volume of distribution (V_d_/F). The exploratory PK parameter estimates obtained through an NCA were suggested as initial PK estimates for PPK modeling. 

Subgroup analysis was performed in patients who initiated their first MMF for aGVHD prophylaxis. The PK parameter estimates were compared between aGVHD patients and non-aGVHD patients in the subgroup analysis. Statistical analysis was performed using the IBM SPSS Statistics (Version 26.0, IBM Corp., Armonk, NY, USA). The Mann–Whitney U test was conducted to determine the difference in AUC_0–6_, AUC_0–inf_, and C_max_ between aGVHD patients and non-aGVHD patients. *p*-value < 0.05 was considered statistically significant. 

#### 2.3.2. Population Pharmacokinetic Analysis

Population pharmacokinetic modeling and analysis were performed using NONMEM^®^ (Version 7.5.0; ICON plc. Dublin, Ireland), Pirana (Version 3.0), and R software (Version 4.2.1). The PPK model was developed using the first-order conditional estimation method with interaction (FOCE-I) to estimate PK parameters. Various disposition models (one and two compartments) and various absorption models (first order with and without lag time, erlang distribution, and transit compartment models) were evaluated as structural models. The model was parameterized with the first-order absorption rate constant (k_a_), V_d_/F, and CL/F. Inter-individual variability was assumed to be log-normal and was assessed using an exponential error model (Equation (1)):

P_i_ = θ × exp(ηP_i_), (1)

where P_i_ is the parameter value for an individual, θ is the typical population value of the parameter, ηP_i_ is the interindividual variability. η was assumed to follow normal distribution around 0 with the variance of ω^2^. The residual error model was described using additive, proportional, and combined (additive and proportional) models. The best structural model and residual error model were selected based on successful minimization status, visual inspection of goodness-of-fit (GOF) plots (including observed (DV) versus individual prediction (IPRED), DV versus population prediction (PRED), conditional weighted residuals (CWRES) versus PRED, and CWRES versus time after the dose), and objective function value (OFV), the value which measures model improvement.

Potential covariates screened on PK parameters were age, sex, height, weight, BSA, underlying hematologic disease, conditioning regimen, donor information, ABO matching, post-transplant days, MMF administration days, MMF formulation, WBC, Hgb, Hct, ANC, BUN, SCr, eGFR, alb, t.bil, AST, ALT, CRP, tacrolimus trough level, and comedications. Before a covariate analysis, correlation between covariates were examined using scatter plots and Pearson correlation coefficients. In cases where covariates showed correlation with correlation coefficients greater than |0.7|, we opted to select only one of the covariates to avoid multicollinearity and instability of PK parameter estimates. Then, the effect of selected covariates on the PK of MMF was assessed using a stepwise covariate selection method (SCM). Forward selection and backward elimination approach were used to determine covariate significance. Covariates were considered statistically significant if the decrease in OFV was >3.84 (*p* < 0.05, degree of freedom (df) = 1) during forward selection and the increase in OFV was >6.63 (*p* < 0.01, df = 1) during backward elimination. The ΔOFV between the two models was assumed to follow the χ^2^ distribution. Then, the final PPK model was developed combining covariates that met the statistical criteria to the structural model. 

### 2.4. Model Evaluation

The predictive performance and stability of the final PPK model was evaluated by internal validation using GOF plots, the bootstrap method, and the visual predictive check (VPC). GOF plots were used to assess the adequacy of fit of the final model. The bootstrap method was performed to assess the robustness of the final model by generating 1000 random resampling datasets and estimating PPK parameter estimates from resampled datasets. The median and the 95% confidence interval (2.5% to 97.5%) of the estimated parameters for 1000 bootstrap replicates were compared to the final model parameter estimates obtained from the original observed dataset. The VPC was used to graphically assess the prediction performance based on 1000 simulated datasets. The 5th, 50th, and 95th percentiles of the observed and simulated data were graphically compared. 

### 2.5. Model Simulation

Using the validated final PPK model, a Monte Carlo simulation involving 1000 iterations was performed to generate PK of MPA under various scenarios including different BSAs (0.5 m^2^, 1.0 m^2^, and 1.5 m^2^) and different dosages of oral administration of MMF (400 mg/m^2^, 600 mg/m^2^, and 900 mg/m^2^ given twice daily). Different doses were derived from the actual dose used at practice based on drug label: 400 mg/m^2^, pediatric dose for kidney transplantation [40]; 600 mg/m^2^, pediatric dose for kidney, heart, and liver transplantation; 900 mg/m^2^, pediatric maximum dose recommended for heart and liver transplantation [41]. Based on the simulation results, the recommended pediatric dose for oral MMF was determined when the AUC_0–12_ were within the therapeutic target range of 30–60 mg·h/L.

## 3. Results

### 3.1. Population Characteristics

A total of 80 total MPA plasma concentrations were collected from 20 pediatric HSCT recipients. The demographic and clinical information of all patients are presented in Table 1. The median age of the patients was 9.7 years (1.7–15.6 years). The diagnoses for HSCT included acute lymphoblastic leukemia, acute myeloid leukemia, aplastic anemia, congenital neutropenia, hemophagocytic lymphohistiocytosis, Krabbe disease, non-Hodgkin lymphoma, and therapy-related myelodysplastic syndrome. The median MMF dosage was 1100 mg/day (17.9 mg/kg/dose). A total of 11 patients (55.0%) developed aGVHD with 4 patients presenting grade III–IV aGVHD. 

### 3.2. Pharmacokinetic Analysis

#### 3.2.1. NCA Results and the Relationship between MMF PK and aGVHD Prophylaxis

**Figure 1:**
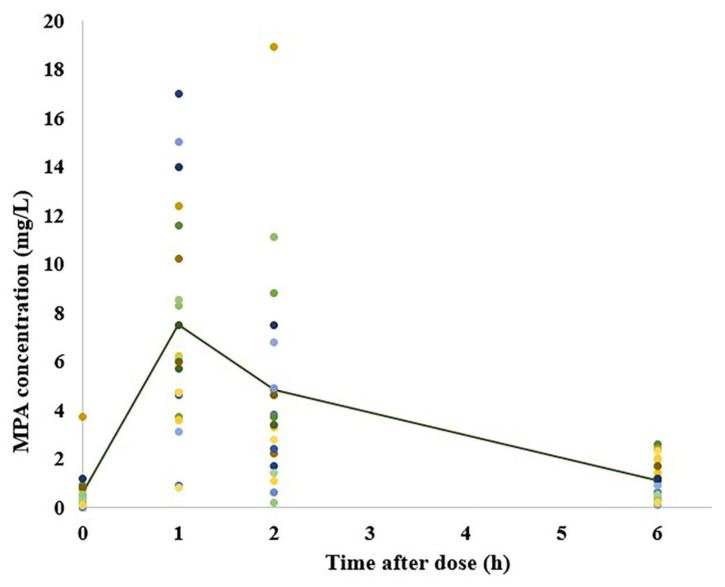
MPA plasma concentration–time profiles of patients. Circles represent individual MPA plasma concentrations. The line represents the mean concentration.

Individual plasma concentration–time profiles for MMF at 0, 1, 2, and 6 h after administration are shown in Figure 1. The estimated PK parameters from an NCA are presented in Table 2. The t_max_ was at 1 h after the MMF administration. 

In the subgroup analysis, among 16 patients who have started their first MMF for aGVHD prophylaxis, aGVHD patients (*n* = 7) demonstrated significantly lower mean AUC_0–6,_ AUC_0–inf_, and C_max_ compared to non-aGVHD patients (*n* = 9) (AUC_0–6_ 13.26 mg·h/L vs. 28.20 mg·h/L, *p* = 0.023), (AUC_0–inf_ 14.78 mg·h/L vs. 35.16 mg·h/L, *p* = 0.016), (C_max_ 5.18 mg/L vs. 10.93 mg/L, *p* = 0.016) (Table 3). Among the seven patients who developed aGVHD in the prophylaxis subgroup patients, five developed grade II aGVHD, and two developed grade III–IV aGVHD. 

#### 3.2.2. Population Pharmacokinetic Analysis

**Figure 2:**
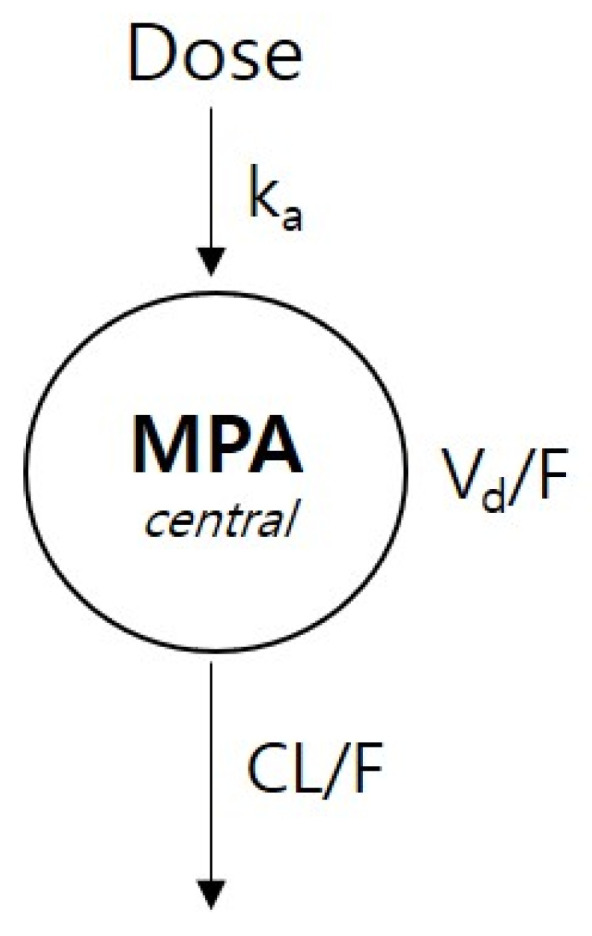
Structure of mycophenolate mofetil population pharmacokinetic model.

A one-compartment model with first-order absorption (ADVAN2 TRANS2 subroutine) was selected as the most suitable structural model to describe the PK of MPA (Figure 2). A combined residual error model was selected to explain the inter-individual variability. The GOF plot is shown in Supplementary Figure S1. Covariate correlation scatterplots with Pearson correlation coefficients for the covariates screening step are provided in Supplementary Figure S2. *BSA* exhibited a statistically significant influence on the V_d_/F of MPA. The addition of this covariate significantly improved the model based on changes in OFV (ΔOFV = −6.175). The final PK parameter estimates for k_a_, V_d_/F, and CL/F were 5.18 h^−1^, 89.8 L, and 16.6 L/h, respectively (Table 4). The final PPK model equation was as follows (Equation (2)):

(2) $$V_{d}/F\left(L\right)=89.83\times \left(1+0.85\;\times \;\left(BSA-1.11\right)\right)\times exp\;(\;\eta_{Vd/F})\;$$

where 1.11 m^2^ was the median value of *BSA* for patients in this study. 

### 3.3. Model Evaluation

**Figure 3:**
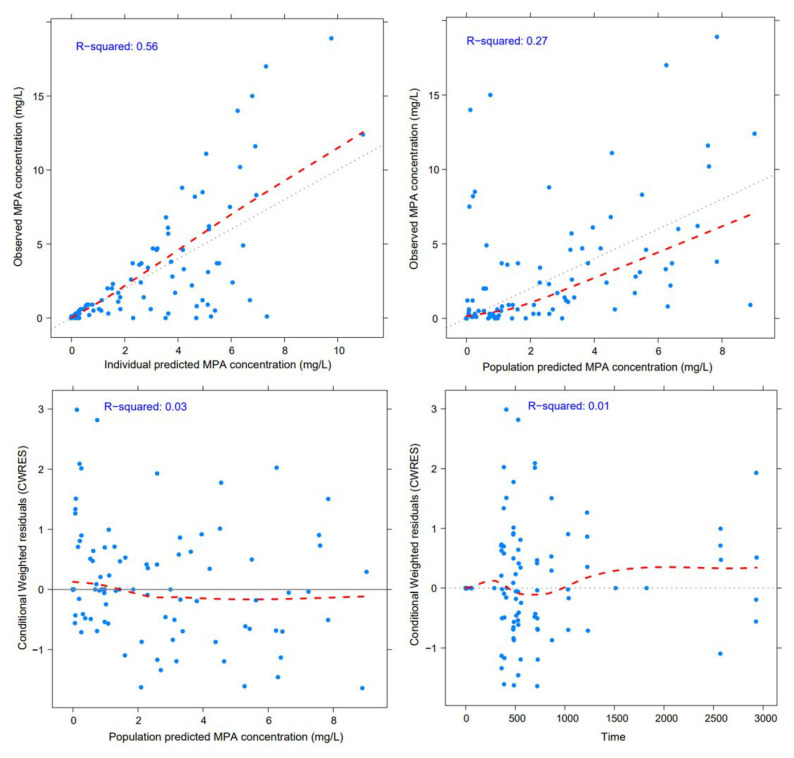
Goodness-of-fit plots of the final model. Blue dots represent MPA plasma concentrations. Gray line represents the line of identity. Red line represents a loess smooth line.

**Figure 4:**
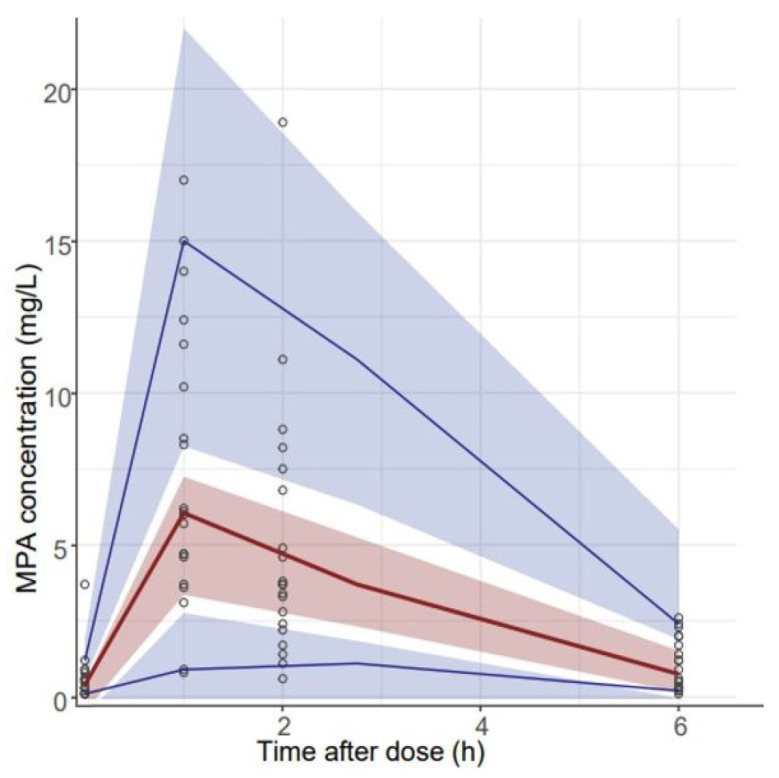
Visual predictive checks of the MPA in the final PPK model. Circles represent the observed concentrations and the red and blue lines represent the observed median and 95% CI. The 95% confidence intervals (CI) for the predicted 5th and 95th percentiles are represented by the blue shaded regions. The 95% CI for the predicted 50th percentiles are represented by the red shaded regions, respectively.

The GOF diagnostic plot of the final model with covariate is shown in Figure 3. Compared to the GOF plot of the structural model, the fitting of GOF plots was slightly improved. PRED and IPRED were in good accordance with the observed concentrations. For CWRES versus PRED and CWRES versus time plots, all points were within −3 and +3 units. The results of the bootstrap method were presented in Table 4. All the PK parameters obtained from the final PPK model were included within the 95% CI of the bootstrap simulation results. The VPC results confirmed the predictive performance of the final PPK model, as the observed concentration data fell within 95% CI and the 5th and 95th percentile range of the simulated data (Figure 4). 

### 3.4. Model Simulation

**Figure 5:**
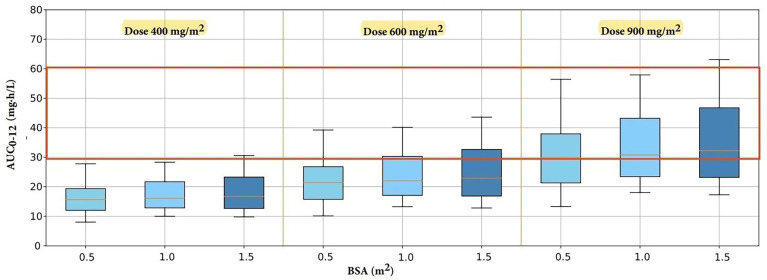
Simulated AUC_0–12_ of MPA with different dosing regimens.

Simulated results are displayed in Figure 5. Based on the simulation results, the recommended dosing regimen for pediatric HSCT recipients to achieve a therapeutic target AUC_0–12_ within the range of 30–60 mg·h/L was the oral administration of MMF at a dose of 900 mg/m^2^ twice daily [11,35,36]. 

## 4. Discussion

In this study, MPA PK in pediatric HSCT recipients was best characterized by a one-compartment model with first-order absorption. BSA was identified as the only covariate with a significant impact on the V_d_/F of MMF. Based on the simulation using the final PPK model, a recommended oral MMF dose of 900 mg/m^2^ twice daily was suggested for pediatric HSCT recipients to achieve the target AUC_0–12_. To the best of our knowledge, this study represents the first PPK study to assess the PK of oral MMF using total MPA concentrations in pediatric HSCT recipients and to recommend a model-informed dosage regimen. 

In the exploratory NCA, the estimated PK parameters were comparable to the PK parameters reported in the previous PPK studies of MMF in pediatric patients [42]. The AUC_0–6_ and AUC_0–inf_ values derived from an NCA were mostly lower than the therapeutic target range of an AUC_0–12_ of 30–60 mg·h/L [11]. The low AUC_0–12_ value can be attributed to various factors that may disrupt drug absorption. These factors, as previously reported in studies, include intestinal mucosal damage resulting from the myeloablative and reduced-intensity conditioning regimen, concurrent antibiotic usage, and concomitant use of proton pump inhibitors in pediatric HSCT patients [22,43,44,45,46]. In this study, 35% of the patients used PPI, which may decrease the MPA concentrations [43]. However, the use of those comedications was not identified as a significant covariate. Furthermore, the statistically significant difference observed in AUC_0–6_, AUC_0–inf_, and C_max_ between aGVHD group and non-aGVHD group among patients who started MMF for aGVHD prophylaxis (*n* = 16), with lower PK parameter estimates in the aGVHD group, highlights the clinical significance of AUC as an important marker of clinical response. Based on the results from previous studies, which indicated that AUC_0–6_ effectively reflects AUC_0–12_ with good correlation (r^2^ = 0.959), our study opted to collect blood samples at 0, 1, 2, and 6 h using a limited sampling strategy and estimate AUC_0–6_ in order to reduce the burden of blood sampling on pediatric patients [47,48].

In the final PPK model, unlike the previous studies involving adult and pediatric transplant recipients, our findings indicate that MPA PK profiles were better described by a one-compartment rather than a two-compartment model [17,18,19,20,21,49]. The PPK estimates for k_a_, V_d_/F, and CL/F were 5.18 h^−1^, 89.8 L, and 16.6 L/h, respectively. The estimated population PK parameters were in a good agreement with the range of reported population-typical values in pediatric solid organ transplant recipients [42]. The k_a_ value in our study fell at the higher end within the previously reported range [42]. This can be explained by the fact that 70% of the patients in our study administered the oral suspension formulation of MMF, which is known for its faster absorption compared to the capsules [18,42]. The MMF formulation was included as one of the screened covariates in this PPK study but had no impact on the PK of MPA. Therefore, it was not included in the final model. Furthermore, covariates included in the previous pediatric studies were weight on CL/F, weight on V_c_/F, comedications, *UGT2B7* variation (802C > T), and weight on CL/F, age on k_a_, post-transplant time on V_d_/F, creatinine clearance (CrCl), t.bil, weight on unbound MPA CL/F, and cyclosporine, weight on CL/F [16,17,19,20,21,49]. Covariates included in the previous adult HSCT PPK studies were serum albumin on CL/F and V_c_/F and CrCl on unbound MPA CL/F [23,25]. Those covariates from previous studies, except genetic variations, were screened in this study, but covariates other than BSA were not included in the final model. This is thought to be due to the characteristics of HSCT patients, who differ from solid organ transplant patients in that they have normal renal and hepatic function. Additionally, this is also believed to be attributed to the limited sample size and limited sampling points in this study pediatric patients. The eta-covariate correlation plot revealed no correlation in other screened covariates. Internal validation was performed using the GOF plot, the bootstrap method, and the VPC, which confirmed the reliability of our population PK estimations and predictive performance in the final PPK model. 

According to the simulation results, the dosing regimen that successfully achieved the therapeutic target AUC_0–12_ of 30–60 mg·h/L, a recognized target for pediatric solid organ transplantation with reported clinical relevance in pediatric HSCT patients, was 900 mg/m^2^ twice daily [11]. In comparison to the pediatric drug label dosage of 600 mg/m^2^ administered twice daily for pediatric solid organ transplantation, our study recommended a higher dosage for pediatric HSCT recipients [41]. Considering that HSCT patients exhibit lower MPA concentrations and AUC_0–12_ levels compared to solid organ transplantation patients, our study suggests that a pediatric MMF dosage of 900 mg/m^2^ administered orally twice daily is an adequate dose for pediatric HSCT patients [11,50]. Several factors, as mentioned earlier, can contribute to low AUC_0–12_ levels in HSCT patients: including HSCT-mediated diarrhea, which can reduce MPA absorption and reabsorption by disrupting the intestinal flora caused by the conditioning regimen and antibiotics [50]. When calculating the dosage based on the body weight and BSA of the study patients, the dosage regimen of 900 mg/m^2^ was approximately 1.5–2-fold higher than the study regimen of 15 mg/kg, suggesting that the study dosage may be insufficient. However, it is important to note that there might be a potential risk of associated adverse drug events when using a higher dosage. Therefore, further research is needed to confirm this recommended dosage regimen in this patient population.

Moreover, in previous pediatric MMF PPK studies in HSCT, Kim et al. [17] developed PPK model on unbound MPA concentration in pediatric and young adult patients undergoing HSCT. However, as total MPA concentrations are generally measured markers in the real clinical practice setting for MPA concentrations, our study developed the PPK model and investigated MPA PK estimates in pediatric HSCT patients using this more commonly employed marker. Zeng et al. [19], which represents the only two pediatric HSCT PPK studies along with Kim et al. [17], investigated MPA PK and evaluated dose regimens in overall pediatric and young adult transplant patients including solid organ transplant and HSCT patients. Hence, the MPA PPK model and recommended dosage regimen developed in this study, taking into account the unique characteristics of pediatric HSCT patients and the real-world clinical practice settings, can serve as a more pragmatic dosage strategy in the actual clinical environment. 

There are several limitations in this study. Firstly, this was a single center study with a limited study sample size. Considering the complex and challenging nature of enrolling pediatric patients, this sample size was recruited to the fullest extent possible during the study period from one of the nationwide high-volume HSCT centers. Thus, a further PPK study with larger sample size is needed in this pediatric population to confirm this study findings. Second, the enterohepatic recirculation (EHR) of MPA-7-O-glucuronide (MPAG) to MPA was not included in the final model. EHR model was investigated in the study PPK modeling, but the model was unsuccessful with minimization terminated due to model instability. Finally, this study did not include genetic polymorphisms as one of the covariates in the PPK model. This was to reflect the actual clinical environment including all the possible patient clinical and laboratory information which is easily accessed in practice. This needs to be further investigated in a future study. 

## 5. Conclusions

In conclusion, this study explicitly assessed the impact of BSA on the population PK characteristics of MPA and suggested a dosage regimen for pediatric HSCT recipients. The model adequately predicted MPA concentrations and AUC_0–12_ in patients with varying BSA and dosing regimens. Simulation supported the need for higher BSA-based dosing regimen in HSCT recipients compared to those undergoing solid organ transplantation. This model-informed optimal dosage regimen and the PPK model for MMF in pediatric HSCT patients can provide a valuable strategy for establishing the appropriate pediatric dosage regimen in real-world clinical practice. However, close monitoring is necessary due to safety concerns arising from higher dosage recommendations.

## Figures and Tables

**Table 1:** Baseline demographic characteristics and clinical information of patients on MMF.

Characteristics	Values
Male gender, *n* (%)	12 (60.0)
Age, years, median (range)	9.7 (1.7–15.6)
1 ≤ age < 12, *n* (%)	14 (70.0)
12 ≤ age < 18, *n* (%)	6 (30.0)
Body weight, kg, median (range)	31.2 (9.9–51.0)
Height, cm, median (range)	136.0 (83.6–176.9)
BSA, m^2^, median (range)	1.12 (0.49–1.60)
Diagnosis for HSCT, *n* (%)	
AA	2 (10.0)
ALL	7 (35.0)
AML	5 (25.0)
Congenital neutropenia	1 (5.0)
HLH	1 (5.0)
Krabbe disease	1 (5.0)
NHL	2 (10.0)
T-MDS	1 (5.0)
Conditioning regimen, *n* (%)	
BuFluATG	1 (5.0)
BuFluCy	15 (75.0)
BuFludaVPATG	1 (5.0)
FluMelATG	1 (5.0)
TBI FluCyATG	2 (10.0)
Donor source, *n* (%)	
Haploidentical family donor/matched unrelated donor	16 (80.0)/4 (20.0)
ABO match, *n* (%)	
Compatible/incompatible	16 (80.0)/4 (20.0)
Patients with previous or concomitant tacrolimus on the day of blood sampling, *n* (%)	20 (100.0)
Previous tacrolimus history, *n* (%)	4 (20.0)
Concomitant tacrolimus, *n* (%)	16 (80.0)
Time post-HSCT, days, median (range) *	31 (20–181)
Duration of MMF, days, median (range) *	23 (16–123)
MMF first use, *n* (%)	
aGVHD prophylaxis/treatment	16 (80.0)/4 (20.0)
MMF dose, mg/day, median (range)	1100 (380–2000)
MMF dose normalized by body weight, mg/kg/dose, median (range)	17.9 (16.1–19.8)
MMF formulation, *n* (%)	
Capsule/suspension	6 (30.0)/14 (70.0)
Laboratory values *	
SCr, mg/dL, median (range)	0.42 (0.32–0.72)
eGFR, mL/min/1.73 m^2^, median (range)	112.6 (86.3–165.3)
AST, IU/L, median (range)	48.5 (26–93)
ALT, IU/L, median (range)	52 (16–251)
T.bil, mg/dL, median (range)	0.5 (0.4–2.0)
Alb, g/dL, median (range)	3.8 (3.3–4.2)
Comedication, *n* (%) *	
Ciprofloxacin	15 (75.0)
Esomeprazole	4 (20.0)
Famotidine	4 (20.0)
Itraconazole	2 (10.0)
Lansoprazole	3 (15.0)
Voriconazole	1 (5.0)

* Values based on the day of blood sampling; *n*, number; BSA, body surface area; HSCT, hematopoietic stem cell transplantation; AA, aplastic anemia; ALL, acute lymphoblastic leukemia; AML, acute myeloid leukemia; HLH, hemophagocytic lymphohistiocytosis; NHL, non-Hodgkin lymphoma; T-MDS, therapy-related myelodysplastic syndrome; BuFluATG, busulfan + fludarabine + anti-thymocyte globulin; BuFluCy, busulfan + fludarabine + cyclophosphamide; BuFludaVPATG, busulfan + fludarabine + etoposide + anti-thymocyte globulin; FluMelATG, fludarabine + melphalan + anti-thymocyteglobulin; TBI, total body irradiation; FluCyATG, fludarabine + cyclophosphamide + anti-thymocyteglobulin; MMF, mycophenolate mofetil; aGVHD, acute graft-versus-host disease; SCr, serum creatinine; eGFR, estimated glomerular filtration rate; AST, aspartate aminotransferase; ALT, alanine transaminase; T.bil, total bilirubin; Alb, albumin.

**Table 2:** Pharmacokinetic parameter estimates based on an NCA.

Pharmacokinetic Parameters	MPA PK Parameter Estimates (Mean ± Standard Deviation) (*n* = 20)
C_max_ (mg/L)	8.50 ± 4.76
AUC_0–6_ (mg·h/L)	22.37 ± 13.50
AUC_0–inf_ (mg·h/L)	27.69 ± 15.94
V_d_/F (L)	69.04 ± 59.58
CL/F (L/h)	23.43 ± 15.69

MPA, mycophenolic acid; PK, pharmacokinetic; *n*, number; C_max_, peak plasma concentration; AUC_0–6_, area under the curve from time 0 to 6 h; AUC_0–inf_, area under the curve from time 0 to infinity; V_d_/F, apparent volume of distribution; CL/F, apparent clearance.

**Table 3:** Pharmacokinetic parameter estimates of patients who have initiated MMF for aGVHD prophylaxis.

Pharmacokinetic Parameters	MPA PK Parameter Estimates *		*p*-Value
	aGVHD Patients (*n* = 7)	Non-aGVHD Patients (*n* = 9)	
C_max_ (mg/L)	5.18 ± 1.09	10.93 ± 5.21	0.016
T_max_ (h)	1.29 ± 0.49	1.44 ± 0.53	0.606
AUC_0–6_ (mg·h/L)	13.26 ± 5.35	28.20 ± 16.64	0.023
AUC_0–inf_ (mg·h/L)	14.78 ± 6.41	35.16 ± 18.86	0.016
V_d_/F (L)	74.55 ± 48.10	54.89 ± 59.13	0.210
CL/F (L/h)	32.69 ± 21.45	18.01 ± 9.50	0.114

* Values are represented as mean ± standard deviation; MPA, mycophenolic acid; PK, pharmacokinetic; aGVHD, acute graft-versus-host disease; *n*, number; C_max_, peak plasma concentration; T_max_, time to reach C_max_; AUC_0–6_, area under the curve from time 0 to 6 h; AUC_0–inf_, area under the curve from time 0 to infinity; V_d_/F, apparent volume of distribution; CL/F, apparent clearance.

**Table 4:** Population pharmacokinetic parameter estimates for MPA in the structural model, final model, and bootstrap.

Parameter	Structural Model (*n* = 20)		Final Model (*n* = 20)		Bootstrap (*n* = 1000)
	Estimate	RSE %	Estimate	RSE %	Median (95% CI *)
Fixed effects					
k_a_ (h^−1^)	6.53	20	5.18	21	5.22 (1.83–7.04)
V_d_/F (L)	77.7	19	89.8	16	85.13 (60.65–121.45)
CL/F (L/h)	16.5	18	16.6	17	17.14 (12.03–23.60)
BSA on V_d_/F	-	-	0.854	24	0.839 (0.32–1.20)
Random effects					
Inter-individual variability (IIV)					
IIV V_d_/F, %CV	49.48	68	37.71	69	35.16 (9.83–62.03)
IIV CL/F, %CV	84.51	33	89.96	33	86.72 (43.83–126.39)
Residual error					
Proportional error	0.675	9	0.660	9	0.656 (0.547–0.763)
Additive error	0.110	24	0.111	17	0.109 (0.083–0.327)

* 2.5th and 97.5th percentiles of bootstrap parameter estimates; RSE, relative standard error; CI, confidence interval; ka, absorption rate constant; V_d_/F, volume of distribution; CL/F, apparent clearance; BSA, body surface area; CV, coefficient of variation (%CV = sqrt(exp(OMEGA) − 1) × 100).

## Data Availability

The raw data supporting the conclusion of this article will be made available by the authors upon reasonable request.

## Supplementary Materials

The following supporting information can be downloaded at: https://www.mdpi.com/article/10.3390/pharmaceutics15122741/s1, Figure S1: Goodness-of-fit plots of a structural model; Figure S2: Correlation matrix of covariates.
