# Supplementary material for: Body Surface Area-Based Dosing of Mycophenolate Mofetil in Pediatric Hematopoietic Stem Cell Transplant Recipients: A Prospective Population Pharmacokinetic Study
Source: Pharmaceutics. 2023 Dec 7;15(12):2741. doi: 10.3390/pharmaceutics15122741 (PMC10748085; doi:10.3390/pharmaceutics15122741)
Supplement: Supplementary file 1 [file pharmaceutics-15-02741-s001.zip › pharmaceutics-2699485-supplementary.pdf]

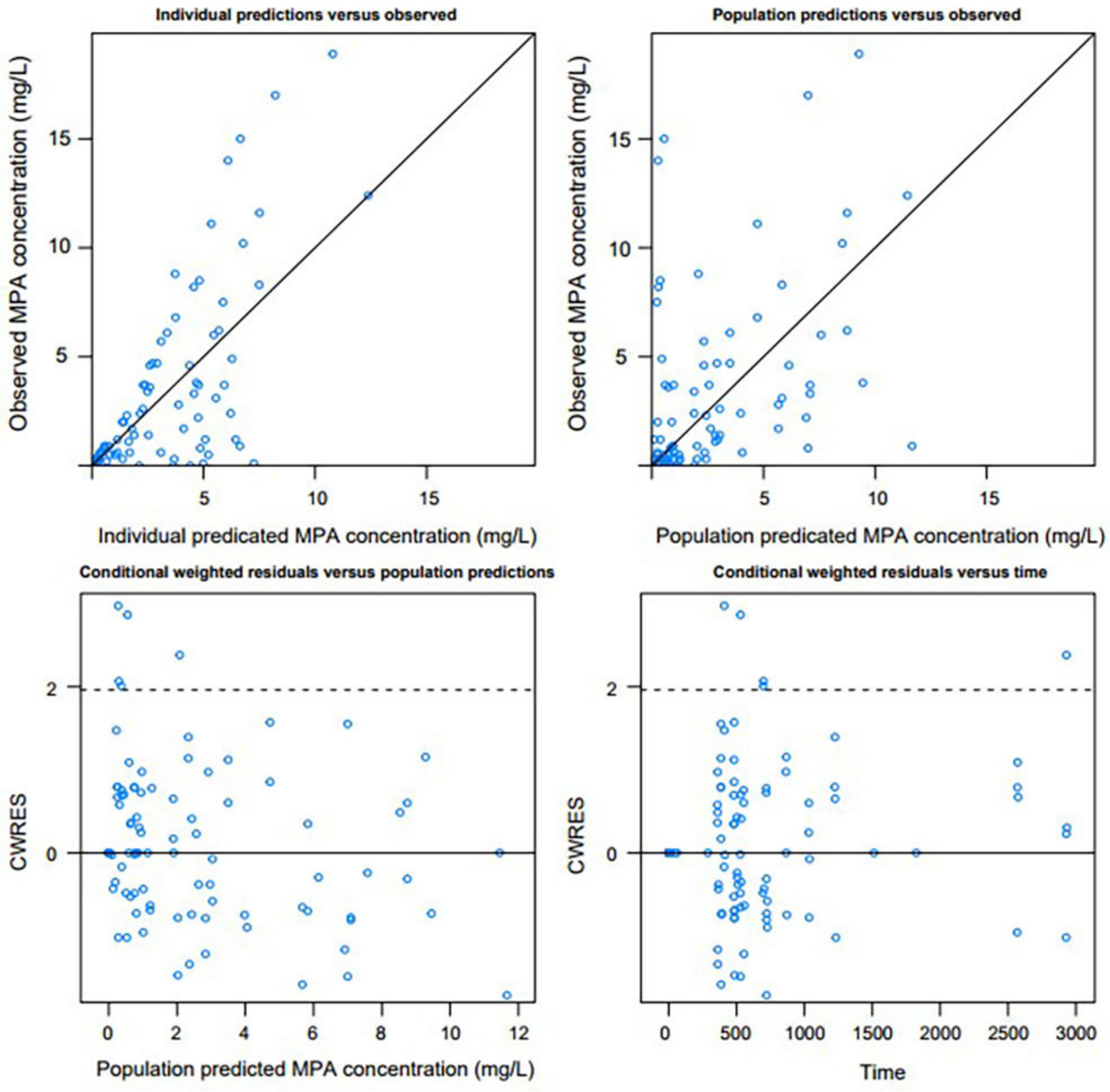

Figure S1. Goodness-of-fit plots of a structural model. MPA, mycophenolic acid; CWRES, conditional weighted residuals.

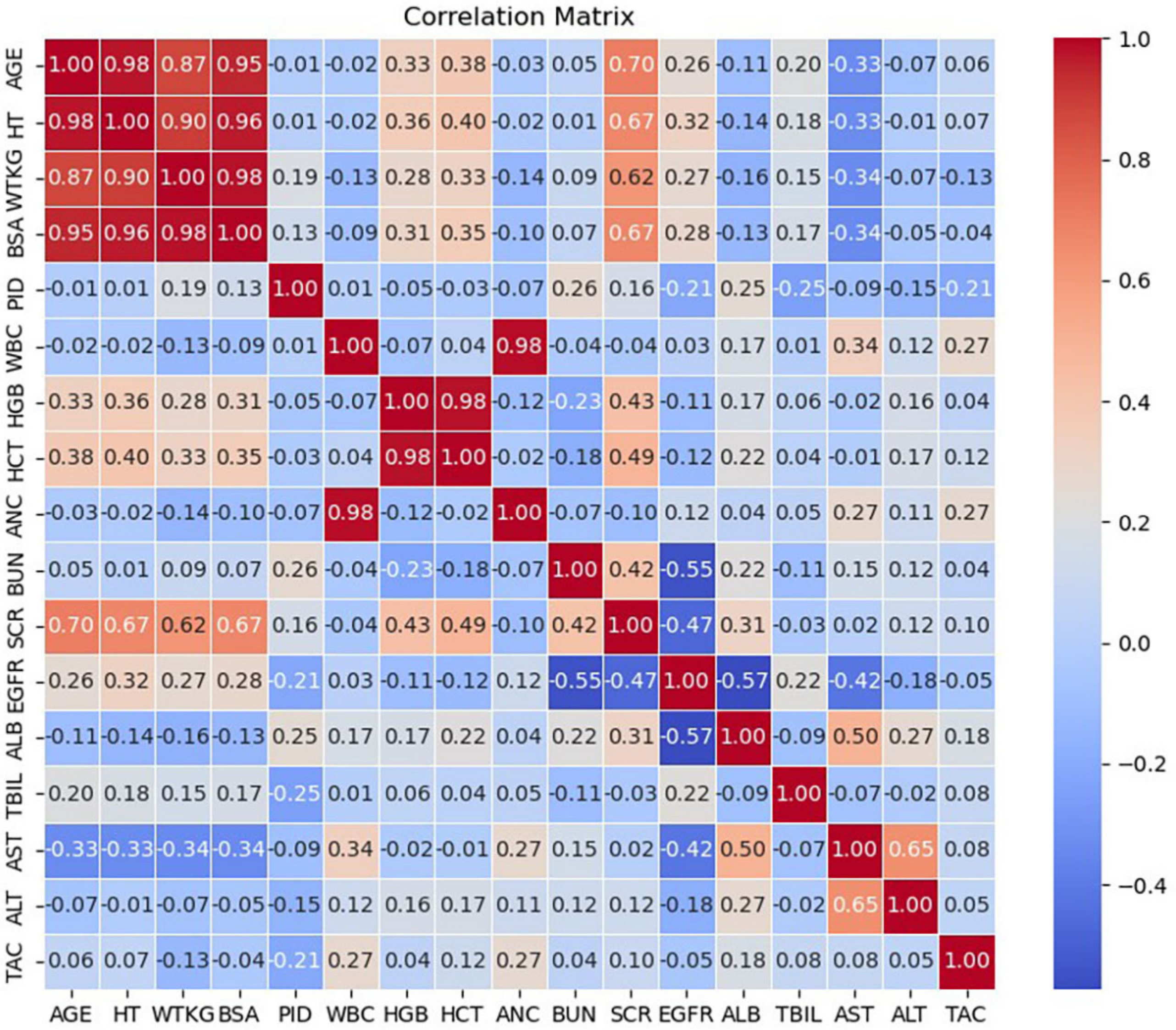

Figure S2. Correlation matrix of covariates. HT, height; WTKG, weight in kilograms; BSA, body surface area; PID, post hematopoietic stem cell infusion days; WBC, white blood cell count; HGB, hemoglobin; HCT, hematocrit; ANC, absolute neutrophil count; BUN, blood urea nitrogen; SCR, serum creatinine; EGFR, estimated glomerular filtration rate; ALB, albumin; TBIL, total bilirubin; AST, aspartate aminotransferase; ALT, alanine transaminase; TAC, tacrolimus trough concentration.
